# Supplementary material for: Optimal Time and Target for Evaluating Energy Delivery after Adjuvant Feeding with Small Bowel Enteral Nutrition in Critically Ill Patients at High Nutrition Risk
Source: Nutrients. 2019 Mar 16;11(3):645. doi: 10.3390/nu11030645 (PMC6470922; doi:10.3390/nu11030645)
Supplement: Supplementary file 1 [file nutrients-11-00645-s001.pdf]

Supplementary Materials

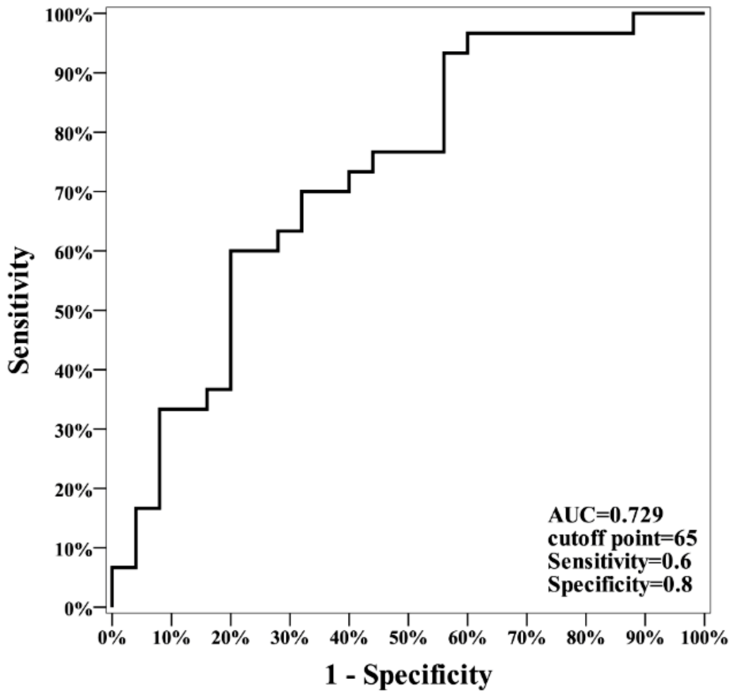

Figure 1. Receiver operating characteristic (ROC) curve to determine the cutoff point for the feeding target between surviving and non-surviving malnourished patients administered SBEN.
